# HLA Haplotype Association with Celiac Disease in Albanian Pediatric Patients from Kosovo

**DOI:** 10.1155/2019/7369014

**Published:** 2019-06-10

**Authors:** Atifete Ramosaj-Morina, Marija Burek Kamenaric, Mehmedali Azemi, Lidvana Spahiu, Zorana Grubic, Renata Zunec

**Affiliations:** ^1^Pediatric Clinic, University Clinical Centre of Kosovo, Rrethi i Spitalit p.n., 10000 Prishtina, Kosovo; ^2^Tissue Typing Centre, Clinical Department of Transfusion Medicine and Transplantation Biology, University Hospital Centre Zagreb, Kispaticeva 12, HR-10000 Zagreb, Croatia

## Abstract

Genetic predisposition to celiac disease (CD) is strongly associated with the presence of HLA alleles in the individual genotype encoding HLA-DQ2 and/or HLA-DQ8 heterodimers. The main aim of this study was to analyze the HLA-A, -B, -DRB1, and -DQ allele and five-locus haplotype frequencies in 60 Albanian pediatric CD patients and 124 non-CD children from Kosovo. The most prevalent haplotype in patients was the ancestral AH 8.1 haplotype present in 22.5% of the cases compared to 2.8% of the controls (*P* < 0.0001). Additionally, two other haplotypes were also overrepresented in patients (HLA-A^∗^02~B^∗^50~DRB1^∗^07~DQA1^∗^02:01~DQB1^∗^02:02 and HLA-A^∗^68~B^∗^44~DRB1^∗^07~DQA1^∗^02:01~DQB1^∗^02:02). Analysis showed that 95.0% of CD patients and 43.3% of controls were carriers of HLA-DQ2 and/or HLA-DQ8 heterodimers. The most frequent CD-predisposing HLA-DQ haplotypes in patients were HLA-DQ2.5 (46.7%) and HLA-DQ2.2 (11.6%), while the most prevalent genotypes were HLA-DQ2.5/DQX (58.3%) and HLA-DQ2.5/DQ2.2 (20.0%). The frequency of the HLA-DQ8 heterodimer among CD patients (4.2%) compared to the control group (8.1%) was without statistical significance. The given data demonstrate differences in the distribution of HLA haplotypes among Albanian CD patients from Kosovo in comparison to other European and non-European populations, as well as provide additional population data to supplement the thus far undisputed importance of the role of HLA-DQ2 and HLA-DQ8 heterodimers in the development of CD.

## 1. Introduction

Celiac disease (CD) is an autoimmune systemic disorder characterized by chronic inflammation of the small intestinal mucosa triggered by gluten and related prolamine uptake that occurs in genetically susceptible individuals [1]. Europe is historically considered a geographical area with a high incidence of CD, with a prevalence of 1%, which may be higher in Northern European countries [2, 3]. Some studies from Scandinavian countries and the United Kingdom population tended to show a higher prevalence of CD of approximately 1.0-1.5% [4]. Although it was thought that some countries, including the United States, were exempt from this disease, it has recently been shown that it has a similar prevalence as in Europe, 0.5-1.0% of the general population [5].

Susceptibility to CD and its activation and perpetuation involve a combination of environmental and genetic factors through some immunological mechanisms [6]. The involvement of Human Leukocyte Antigens (HLA) in CD pathogenesis was first described in the 1970s [7]. Over the subsequent years, the specific alleles that underlie the described associations became clear: CD is strongly associated with HLA-DQ, which encodes HLA-DQ2 and HLA-DQ8 heterodimers.

At least 90% of the patients with CD are positive for the HLA-DQ2 heterodimer in *cis* formation (HLA-DQ2*cis*) encoded by HLA-DQA1^∗^05:01 (*α*-chain) and DQB1^∗^02:01 (*β*-chain) alleles on a HLA-DRB1^∗^03 haplotype, although the HLA-DQ2 heterodimer may also be encoded in *trans* position (HLA-DQ2*trans*) with the HLA-DQA1^∗^05:05 allele, usually on HLA-DRB1^∗^11, DRB1^∗^12, DRB1^∗^13:03, and DRB1^∗^13:05 haplotypes, and the HLA-DQB1^∗^02:02 allele, usually on a HLA-DRB1^∗^07 haplotype [8, 9], but in some populations on HLA-DRB1^∗^04 haplotypes as well. The majority of the remaining CD patients carry the HLA-DQ8 heterodimer formed by one *α*-chain and one *β*-chain encoded with HLA-DQA1^∗^03:01 and HLA-DQB1^∗^03:02 alleles on the HLA-DRB1^∗^04 haplotypes [10] and sporadically on HLA-DRB1^∗^08 haplotypes.

Most of the studies exploring the linkage of HLA genes with CD are focused on HLA class II genes encoding HLA-DQ heterodimers, while very few studies investigate extended HLA haplotypes [11]. In contrast to a relatively conserved HLA-DR3 haplotype, the linkage between HLA loci forming the HLA-DR7 and HLA-DR11 haplotypes are not so strong, and since they can form the HLA-DQ2.5*trans* heterodimer, we wanted to explore if there is a difference in HLA haplotype distribution among CD patients in comparison to healthy subjects.

The importance of genetic factors in the pathogenesis of CD is well documented by many studies. It is clear that CD rarely develops in the absence of HLA-DQ2 and/or HLA-DQ8 heterodimers, and that the predisposing HLA-DQ2 and HLA-DQ8 subtypes are necessary, but not sufficient for causing the disease [12]. Anderson et al. suggested that a combination of HLA typing and confirmatory serology could reduce the number of unnecessary endoscopies as well as the number of false negatives and/or positive diagnoses [13]. In 2012, the role of HLA in the diagnosis of CD was firmly recognized, which resulted in important changes in diagnostic criteria and the inclusion of HLA typing in CD diagnostic guidelines [1].

As far as we know, no previous study has focused on the frequency of CD-predisposing HLA genotypes in affected and nonaffected individuals in Kosovar Albanian children. Thus, the main aim of this research was to analyze the HLA-A~B~DRB1~DQA1~DQB1 haplotype distribution as well as the frequency of CD-predisposing HLA-DQ genotypes in Albanian pediatric CD and non-CD subjects from Kosovo.

## 2. Materials and Methods

### 2.1. Patients and Control Group

Sixty pediatric patients with CD (40 females and 20 males) at the University Clinical Centre of Kosovo (UCCK) and 124 unrelated age- and gender-matched children (64 females and 60 males) without a history of autoimmune diseases were included in this study. The patients' age at time of diagnosis ranged from 17 months to 18 years, with a mean age of 5.5 years (SD ± 3.31). The control group age at the time of sample collection ranged from 1 to 18 years with the mean age of 8.7 years (SD ± 5.8). The CD diagnosis was achieved according to the criteria of the European Society of Pediatric Gastroenterology and Nutrition [14] and the revised guidelines of the European Society of Pediatric Gastroenterology, Hepatology, and Nutrition [1]. The study was approved by the Ethics Committee of the UCCK (approved February 12th, 2013) and by the Ethics Committee of the University Hospital Centre (UHC) Zagreb (approved March 28th, 2013) and is in accordance with the Helsinki Declaration. Written informed consent was obtained from parents or caregivers of all children prior to their enrollment in the study.

### 2.2. HLA Typing

Three milliliters of EDTA blood samples were collected from each child included in the study. Blood samples were stored frozen at -30°C until all samples were collected. DNA isolation and HLA typing were performed in the Tissue Typing Centre, Department of Transfusion Medicine and Transplantation Biology, UHC Zagreb. Genomic DNA was isolated from whole blood with the MagNA Pure Compact Instrument using the corresponding commercially available MagNA Pure Compact Nucleic Acid Isolation Kit I (Roche Diagnostics GmbH, Mannheim, Germany). The final DNA concentration was adjusted to 50 ng/*μ*l. All CD patients and controls were typed for HLA-A, -B, -DRB1, -DQA1, and -DQB1 applying the standard polymerase chain reaction–sequence-specific oligonucleotide probing (PCR-SSOP) method using the commercially available Immucor LIFECODES HLA-SSO typing kit (Immucor Transplant Diagnostics Inc., Stamford, USA), based on the hybridization of a labeled single-stranded PCR product to SSO probes [15]. After hybridization, the samples were processed and quantified on the Luminex 200 flow analyzer (Luminex Corporation, Austin, USA) and the resulting data were analyzed with Match It! DNA Software, version 1.2.4. The HLA-DQA1 and -DQB1 alleles determined at medium resolution level were assigned to the most common corresponding four-digit HLA allele which was additionally confirmed when necessary by the standard polymerase chain reaction–sequence-specific primer (PCR-SSP) high-resolution protocol (CareDx, Olerup SSP AB, Sweden).

### 2.3. Statistical Analysis

The observed HLA-A, -B, -DRB1, -DQA1, and -DQB1 frequencies in both research groups and five-locus haplotype estimates were calculated using the PyPop (Python for Population genetics, version 0.7.0; http://www.pypop.org) program [16].

Regarding the HLA-DQ profile of the subjects, they were categorized according to the presence of susceptible haplotypes (DQ2.5*cis*, DQ2.5*trans*, and DQ8), fractional susceptible haplotypes (DQ2.2, DQ2.3, and DQ7), or nonsusceptible haplotypes (DQ4, DQ5, DQ6, and DQ9). Additionally, based on the copy number of the CD-predisposing HLA-DQ2 and/or HLA-DQ8 alleles in each individual, dual-dosage susceptible genotypes (DQ2/DQ2, DQ2/DQ8, and DQ8/DQ8), sole-dosage susceptible genotypes (DQ2/DQX or DQ8/DQX), and nonsusceptible (DQ2 and/or DQ8 negative) genotypes were defined. Fisher's exact test was applied for comparisons between the HLA allelic groups, haplotypes, and genotypes of CD patients and those of the control group, and calculated differences were considered statistically significant if the *P* value was less than 0.05.

## 3. Results

### 3.1. HLA Allele Frequencies

The observed HLA allele frequencies of HLA-A, -B, -DRB1, -DQA1, and -DQB1 loci with a statistically significant difference between the Albanian pediatric CD patients and the control group from Kosovo are shown in Table 1 and Table 2.

A total of 15 HLA-A alleles were found in the CD patient group, among which the most frequent were HLA-A^∗^01 (26.7%), -A^∗^02 (21.7%), and -A^∗^03 (10.0%). A statistically significant difference was observed for HLA-A^∗^01 (*P* < 0.0001), present with a higher frequency among CD patients compared to the control group, while the HLA-A^∗^02 allele had a lower frequency among CD patients (*P* = 0.0312).

At the most polymorphic HLA-B loci with 21 different alleles detected in the CD patient group, seven alleles showed a frequency > 5.0%: HLA-B^∗^07 (8.3%), -B^∗^08 (29.2%), -B^∗^18 (6.7%), -B^∗^38 (5.0%), -B^∗^44 (8.3%), -B^∗^50 (5.8%), and -B^∗^51 (10.8%). A significantly higher frequency of HLA-B^∗^08 (*P* < 0.0001) and HLA-B^∗^50 (*P* = 0.0012) as well as a significantly lower frequency of HLA-B^∗^35 (*P* = 0.0093) was detected among CD patients in comparison to the control group.

Out of the 12 different alleles observed at the HLA-DRB1 locus among CD patients, the three most frequent were HLA-DRB1^∗^03 (38.3%), -DRB1^∗^07 (17.5%), and -DRB1^∗^11 (13.3%). Additionally, HLA-DRB1^∗^03 and -DRB1^∗^07 are alleles with a much higher frequency among CD patients than in the control group (*P* < 0.0001 and *P* = 0.0023, respectively), as opposed to HLA-DRB1^∗^11, -DRB1^∗^13, and -DRB1^∗^16 with a significantly lower frequency among CD patients (*P* = 0.0460, *P* = 0.0102, and *P* = 0.0289, respectively).

At the HLA-DQ locus, 10 different HLA-DQA1 and 12 different HLA-DQB1 alleles were observed in CD subjects. The most frequent HLA-DQA1 alleles were HLA-DQA1^∗^01:02 (13.3%), -DQA1^∗^02:01 (17.5%), -DQA1^∗^05:01 (40.0%), and -DQA1^∗^05:05 (13.3%), with a statistically significant difference and higher frequency for HLA-DQA1^∗^02:01 (*P* = 0.0023) and -DQA1^∗^05:01 (*P* < 0.0001) in comparison to the control group. On the other hand, the frequency of the HLA-DQA1^∗^01:02 (*P* = 0.0049) and -DQA1^∗^05:05 (*P* = 0.0115) alleles is lower among CD patients. HLA-DQB1^∗^02:01 (40.0%), -DQB1^∗^02:02 (18.3%), and -DQB1^∗^03:01 (14.2%) were the most frequent alleles of the HLA-DQB1 locus in the CD patient group, and a significantly higher frequency in the CD patient group in comparison to controls was observed just for HLA-DQB1^∗^02:01 (*P* < 0.0001) and -DQB1^∗^02:02 (*P* = 0.0020). On the other hand, the frequencies of HLA-DQB1^∗^03:01 and -DQB1^∗^05:02 alleles were significantly lower among CD patients (*P* = 0.0084 and *P* = 0.0341, respectively) in comparison to the control group.

### 3.2. HLA-A~B~DRB1~DQA1~DQB1 Haplotype Frequencies

A total of 71 different five-locus haplotypes (HLA-A~B~DRB1~DQA1~DQB1) were detected in the CD patient group with a total of 55 haplotypes that occurred only once. The most prevalent haplotype observed was HLA-A^∗^01~B^∗^08~DRB1^∗^03~DQA1^∗^05:01~DQB1^∗^02:01 (22.5%) followed by HLA-A^∗^02~B^∗^50~DRB1^∗^07~DQA1^∗^02:01~DQB1^∗^02:02 and HLA-A^∗^02~B^∗^51~DRB1^∗^11~DQA1^∗^05:05~DQB1^∗^03:01 with a frequency of 4.1%, each. When comparing the CD patients' haplotype frequency results to the frequencies of 156 different five-locus haplotypes observed in the control group (Table 3), a statistically significant difference was calculated for the HLA-A^∗^01~B^∗^08~DRB1^∗^03~DQA1^∗^05:01~DQB1^∗^02:01 (*P* < 0.0001) and HLA-A^∗^02~B^∗^50~DRB1^∗^07~DQA1^∗^02:01~DQB1^∗^02:02 (*P* = 0.0311) haplotypes, both more frequent among CD patients. Also, the HLA-A^∗^68~B^∗^44~DRB1^∗^07~DQA1^∗^02:01~DQB1^∗^02:02 haplotype was observed with a high frequency (3.3%) among the CD patient group, which resulted in a significant difference (*P* = 0.0049) due to a 0% frequency of this haplotype in the control group.

### 3.3. Frequencies of CD-Predisposing HLA-DQ Haplotypes and Genotypes

The presence of the HLA-DQ2 and/or HLA-DQ8 heterodimers was detected in 57/60 (95.0%) CD patients and in 50/124 (40.3%) control subjects. The detailed CD-predisposing HLA-DQ haplotype and genotype distribution among CD patients and a comparison with the frequency of those haplotypes/genotypes in the control group is presented in Table 4. The most frequent CD-predisposing HLA-DQ haplotypes found in patients were HLA-DQ2.5 (*cis* and *trans* conformation) and HLA-DQ2.2 with a frequency of 46.7% and 11.6%, respectively. Consequently, the most prevalent genotype among CD patients was HLA-DQ2.5/DQX with a frequency of 58.3%, followed by the HLA-DQ2.5/DQ2.2 genotype with a frequency of 20.0%. When compared with the control group, both genotypes show a statistically significantly higher frequency among CD patients (*P* < 0.0001 and *P* = 0.0005, respectively). Three patients were homozygous for HLA-DQ2.5 (having a genotype positive for two copies of the HLA-DQ2.5 heterodimer), with no presence of this genotype in controls. The frequency of the HLA-DQ8 haplotype among CD patients is low (4.2%), and there is no significant difference compared to the control group (8.1%). Looking at the HLA-DQ8-related genotypes, 3 (5.0%) CD patients and 1 (0.8%) individual from the control group carried HLA-DQ8/DQ2.5, while 2 (3.3%) CD patients, but as many as 17 (13.7%) individuals from the control group, carried the HLA-DQ8/DQX genotype (*P* = 0.0459). Only one individual in the control group had the HLA-DQ8 genotype alone (DQ8/DQ8). Three CD patients who are not carriers of any of the susceptible variants (HLA-DQ2.5, HLA-DQ2.2, or HLA-DQ8) had the HLA-DQ7/DQ7, HLA-DQ7/DQ5, and HLA-DQ5/DQ6 genotypes.

## 4. Discussion

A strong association between the CD and HLA-DQ allelic groups encoding for HLA-DQ2 and HLA-DQ8 heterodimers is well-described, and these specific HLA heterodimers are usually seen in more than 90.0% of the patients with CD [17]. However, due to the huge polymorphism of the HLA system and the different HLA risk levels of CD among populations [18], it is important to perform the analysis in each specific population separately. This is the first analysis of the HLA-A, -B, -DRB1, -DQA1, and -DQB1 allele frequencies and the HLA-DQ2 and HLA-DQ8 heterodimer frequencies in 60 Albanian children from Kosovo diagnosed with CD. The limitation of the study is the small sample size, although the post hoc sample size calculation showed that the number of 60 CD patients was enough for the obtained results with a confidence interval of 85%.

The analysis of the HLA-A, -B, -DRB1, and -DQ frequencies among CD patients and compared to the control group revealed a pronounced increase of the HLA-A^∗^01, -B^∗^08, -B^∗^50, -DRB1^∗^03, -DRB1^∗^07, -DQA1^∗^02:01, -DQA1^∗^05:01, -DQB1^∗^02:01, and DQB1^∗^02:02 alleles. On the other hand, HLA-A^∗^02, -B^∗^35, -DRB1^∗^11, -DRB1^∗^13, -DRB1^∗^16, -DQA1^∗^01, DQB1^∗^05, and -DQB1^∗^03:01 were significantly less present among CD patients than in controls. The higher frequency of these HLA alleles among CD patients is also reflected in the haplotype frequency. The top-ranked haplotype observed with a high frequency of 22.5% was the ancestral AH 8.1 haplotype (HLA-A^∗^01~B^∗^08~DRB1^∗^03~DQA1^∗^05:01~DQB1^∗^02:01), which was by contrast present in 2.8% of controls. These results are in concordance with results showing that DQ2 is an absolute requirement for the development of CD, but the presence of the well-known ancestral haplotype AH 8.1 and additional genetic factors in the HLA class I region induce an increased risk of CD [19–22].

What is also interesting are the two HLA alleles with a surprisingly high frequency in the CD patient group: HLA-B^∗^50 and HLA-DRB1^∗^07, with frequencies that were more than four times higher and three times higher in comparison to controls, respectively. Consequently, the second most frequent five-locus haplotype observed in our CD patients was HLA-A^∗^02~B^∗^50~DRB1^∗^07~DQA1^∗^02:01~DQB1^∗^02:02. This haplotype has not been previously reported as a haplotype associated with CD, and it is mostly associated with a probable Euro-Asiatic origin, having been reported in Mongolians (HF: 3.2%), in the Chaouya population from Morocco (HF: 2.9%), Turks and Kurds (HF: 1.3%), Spaniards (HF: 1.2%), and Italians (HF: 0.5%) [23–25]. The second uncommon and unexpectedly frequent haplotype, ranked fourth among our CD patients, was HLA-A^∗^68~B^∗^44~DRB1^∗^07~DQA1^∗^02:01~DQB1^∗^02:02. This haplotype was not observed in the control group, and this five-locus haplotype has not been reported in any European population so far, but only in the Sri Lanka Colombo population with a frequency of 7.0% [26].

These results raise new questions: what is the distribution of this five-locus HLA haplotype in other neighboring populations as well as in other populations of European origin? Furthermore, is the presence of this haplotype specific just for Albanian CD patients from Kosovo, or is this haplotype present among CD patients from other countries?.

The results of this research revealed that 95.0% of the Albanian pediatric CD patients from Kosovo were HLA-DQ2- and/or HLA-DQ8-positive compared to the 40.3% of positive individuals in the control group. The given results are similar to those reported from other studies, and at the same time they confirm the variability of HLA-DQ2 and HLA-DQ8 heterodimer frequencies among CD patients from different populations. One large European collaborative study comprising 1008 CD patients from Finland, France, Italy, Norway, Sweden, and the UK showed that HLA-DQ2 and HLA-DQ8 heterodimer frequencies were higher in the Northern European population (Finland—96.0%, Norway + Sweden—96.6%, UK—95.7%) than in the Southern European population (France—93.4% and Italy—89.5%) [27]. Since Kosovo is situated in Southeastern Europe, our result of 95.0% DQ2- and DQ8-positive patients is in concordance with this observation. Furthermore, our results are very similar to results from Greece where a single centre study found that 95.8% of pediatric CD patients were HLA-DQ2- and/or HLA-DQ8-positive in comparison to 32.5% of healthy individuals [28]. On the other hand, a study from Croatia reports a much higher frequency (98.0%) of patients with CD who were carriers of HLA-DQ2 and HLA-DQ8 heterodimers [29].

Epidemiological HLA studies have shown that the HLA-DQ gene dose has a strong quantitative effect on the magnitude of gluten-specific T cell responses, and these individuals have the highest risk of developing CD [10]. In the present study, 18 (30.0%) patients had a dual dosage of HLA-DQ-susceptible genotypes; 12 were HLA-DQ2.5/2.2 heterozygous, 3 HLA-DQ2.5 homozygous, and 3 were HLA-DQ2/DQ8 heterozygous. The majority of HLA-DQ2-positive patients were found to be homozygous or heterozygous for the HLA-DRB1^∗^03~DQA1^∗^05:01~DQB1^∗^02:01 haplotype (HLA-DR3~DQ2), so the likelihood of HLA-DQ2 positivity (91.6%) is in line with reports from the European populations (90.0-95.0%) [18, 23]. Interestingly, the incidence of the HLA-DQ8 heterodimer (4.2%) alone, in double dose or in combination with other heterodimers, was lower in our CD cases than in controls, but with no statistically significant difference. Those result are in line with the studies from different European populations reporting the incidence of the HLA-DQ8 heterodimer among CD patients from 2.8% to 7.9% [27, 29, 30], although there are also studies reporting much higher HLA-DQ8 heterodimer frequencies, even up to 25.0% [28, 31].

In our cohort, 5.0% (3/60) of CD patients were HLA-DQ2- and/or HLA-DQ8-negative, which is comparable to the data originating from different countries reporting the percentage of HLA-DQ2/DQ8-negative CD patients between 0 and 10.0% [27, 29, 30, 32]. Two out of three HLA-DQ2/DQ8-negative CD patients were positive for the DQA1 part of the DQ2 heterodimer, carrying the HLA-DRB1^∗^11~DQA1^∗^05:05~DQB1^∗^03:01 haplotype. The third patient was negative for all CD-predisposing DQ alleles (HLA haplotypes: HLA-DRB1^∗^13~DQA1^∗^01:03~DQB1^∗^06:03 and HLA-DRB1^∗^15~DQA1^∗^01:02~DQB1^∗^05:02), with positive serology and partial villous atrophy who responded to a gluten-free diet.

In conclusion, this study supports the idea that possibly, in addition to the well-known association of CD with HLA class II alleles, extended HLA haplotypes might be considered as a potential genetic risk factor. The given results also provide population data of Albanian CD patients from Kosovo and support the importance of HLA-DQ2 and HLA-DQ8 heterodimers in the development of CD.

## Figures and Tables

**Table 1:** The HLA-A, -B, and -DRB1 allele frequencies observed in the Albanian pediatric celiac disease patients from Kosovo (*N* = 60) with statistically significant differences in comparison to healthy individuals in the control group (*N* = 124).

	Patients (*N* = 60)		Control group (*N* = 124)		
	*n*	AF	*n*	AF	*P*
HLA-A^∗^					
01	32	0.2667	26	0.1048	**<0.0001**
02	26	0.2167	81	0.3266	**0.0312**
HLA-B^∗^					
08	35	0.2917	14	0.0565	**<0.0001**
35	4	0.0333	31	0.1250	**0.0093**
50	7	0.0583	1	0.0040	**0.0012**
HLA-DRB1^∗^					
03	46	0.3833	18	0.0726	**<0.0001**
07	21	0.1750	17	0.0685	**0.0023**
11	16	0.1333	55	0.2218	**0.0460**
13	6	0.0500	36	0.1452	**0.0102**
16	7	0.0583	34	0.1371	**0.0289**

Legend (alphabetic order): AF = allele frequency; *N* = number of tested individuals; *n* = number of allelic group occurrence.

**Table 2:** The HLA-DQA1 and -DQB1 allele frequencies observed in the Albanian pediatric celiac disease patients from Kosovo (*N* = 60) with statistically significant differences in comparison to healthy individuals in the control group (*N* = 124).

	Patients (*N* = 60)		Control group (*N* = 124)		
	*n*	AF	*n*	AF	*P*
HLA-DQA1^∗^					
01:02	16	0.1333	66	0.2661	**0.0049**
02:01	21	0.1750	17	0.0685	**0.0023**
05:01	48	0.4000	19	0.0766	**<0.0001**
05:05	16	0.1333	62	0.2500	**0.0115**
HLA-DQB1^∗^					
02:01	48	0.4000	18	0.0726	**<0.0001**
02:02	22	0.1833	19	0.0766	**0.0020**
03:01	17	0.1417	66	0.2661	**0.0084**
05:02	8	0.0667	36	0.1452	**0.0341**

Legend (alphabetic order): AF = allele frequency; *N* = number of tested individuals; *n* = number of allele occurrence.

**Table 3:** The HLA-A~B~DRB1~DQA1~DQB1 haplotypes with ≥2 number of haplotype copies in the Albanian pediatric celiac disease patients from Kosovo (*N* = 60) and compared with the frequencies of those haplotypes in the control group (*N* = 124).

Haplotype HLA	Patients (*N* = 60)		Control group (*N* = 124)		
A^∗^~B^∗^~DRB1^∗^~DQA1^∗^~DQB1^∗^	*n*	HF	*n*	HF	*P*
01**~**08**~**03**~**05:01**~**02:01	27.0	0.2250	7.0	0.0282	**<0.0001**
02**~**50**~**07**~**02:01**~**02:02	5.0	0.0416	1.0	0.0041	**0.0311**
02**~**51**~**11**~**05:05**~**03:01	5.0	0.0416	6.0	0.0242	0.3619
68**~**44**~**07**~**02:01**~**02:02	4.0	0.0334	0	0	**0.0049**
24**~**07**~**15**~**01:02**~**06:02	3.0	0.0250	3.0	0.0121	0.3699
23**~**18**~**11**~**05:05**~**03:01	3.0	0.0250	0	0	0.0755
68**~**40**~**03**~**05:01**~**02:01	2.0	0.0167	1.0	0.0041	0.2443
02**~**13**~**07**~**02:01**~**02:02	2.0	0.0167	1.0	0.0041	0.2443
02**~**08**~**03**~**05:01**~**02:01	2.0	0.0167	1.0	0.0041	0.2443
03**~**44**~**07**~**02:01**~**02:02	2.0	0.0167	1.0	0.0041	0.2443
24**~**08**~**03**~**05:01**~**02:01	2.0	0.0167	0	0	0.1303
32**~**08**~**03**~**05:01**~**02:01	2.0	0.0167	1.0	0.0041	0.2443
03**~**38**~**13**~**01:03**~**06:03	2.0	0.0167	3.0	0.0121	0.7237
29**~**27**~**11**~**05:01**~**02:01	2.0	0.0167	1.0	0.0041	0.2443
23**~**44**~**07**~**02:01**~**02:02	2.0	0.0167	6.0	0.0242	0.6444
03**~**07**~**03**~**05:01**~**02:01	2.0	0.0167	0	0	0.1303

Legend (alphabetic order): HF = haplotype frequency; *N* = number of tested individuals; *n* = number of observed haplotypes.

**Table 4:** The frequency of celiac disease-predisposing HLA-DQ haplotypes and genotypes detected among Albanian pediatric celiac disease patients from Kosovo (*N* = 60) and the control group (*N* = 124).

	Patients (*N* = 60)	Control group (*N* = 124)	
HLA-DQ haplotypes	*n* (%)	*n* (%)	*P*
Susceptible haplotypes			
DQ2.5*cis*	48 (40.01)	19 (7.66)	**<0.0001**
DQ2.5*trans*	8 (6.66)	3 (1.20)	**0.0088**
DQ8	5 (4.17)	20 (8.06)	0.1712
Fractional susceptible haplotypes			
DQ2.2	14 (11.66)	14 (6.45)	0.0631
DQ2.3	0	1 (0.41)	0.8170
DQ7	17 (14.16)	67 (27.01)	**0.0068**
Nonsusceptible haplotypes			
DQ4	1 (0.83)	6 (2.41)	0.3191
DQ5	15 (12.50)	68 (27.41)	**0.0017**
DQ6	12 (10.00)	48 (19.35)	**0.0252**
DQ9	0	2 (0.80)	0.5650
HLA-DQ genotypes			
DQ2 and/or DQ8 positive	57 (95.00)	50 (40.32)	**<0.0001**
Dual-dosage susceptible genotypes			
DQ2.5/DQ2.2	12 (20.00)	2 (1.61)	**0.0005**
DQ2.5/DQ2.5	3 (5.00)	0	0.0738
DQ2.2/DQ2.2	0	2 (1.61)	0.5616
DQ8/DQ2.5	3 (5.00)	1 (0.81)	0.1091
DQ8/DQ2.2	0	0	n/a
DQ8/DQ8	0	1 (0.81)	0.8144
Sole-dosage susceptible genotypes			
DQ2.5/DQX	35 (58.33)	19 (15.32)	**<0.0001**
DQ2.2/DQX	2 (3.33)	8 (6.45)	0.3902
DQ8/DQX	2 (3.33)	17 (13.71)	**0.0459**
DQ2 and/or DQ8 negative	3 (5.00)	74 (59.68)	**<0.0001**

Legend (alphabetic order): DQ2.5 = HLA-DQA1^∗^05~DQB1^∗^02 (HLA-DRB1^∗^03); DQ8 = HLA-DQA1^∗^03~DQB1^∗^03:02 (HLA-DRB1^∗^04); DQ2.2 = HLA-DQA1^∗^02~DQB1^∗^02 (HLA-DRB1^∗^07); DQ2.3 = HLA-DQA1^∗^03~DQB1^∗^02 (HLA-DRB1^∗^04/^∗^09/^∗^11); DQ7 = HLA-DQB1^∗^03:01 (HLA-DRB1^∗^11/^∗^12/X); DQ4, DQ5, DQ6, and DQ9 were assigned if HLA-DQB1^∗^04, HLA-DQB1^∗^05, HLA-DQB1^∗^06, and HLA-DQB1^∗^03:03 alleles were present; DQX = presence of any other HLA-DQB1 allele than HLA-DQ2 and/or HLA-DQ8; *N* = number of tested individuals; *n* = number of HLA-DQ haplotype/genotype occurrence; n/a = not applicable (due to *n* = 0).

## Data Availability

The HLA data used to support the findings of this study are available from the corresponding author upon request.
